# The Clinicopathological Significance of BiP/GRP-78 in Breast Cancer: A Meta-Analysis of Public Datasets and Immunohistochemical Detection

**DOI:** 10.3390/curroncol29120710

**Published:** 2022-11-23

**Authors:** Inês Direito, Daniela Gomes, Fátima Liliana Monteiro, Isa Carneiro, João Lobo, Rui Henrique, Carmen Jerónimo, Luisa Alejandra Helguero

**Affiliations:** 1 iBiMED—Institute of Biomedicine, University of Aveiro, Agra do Crasto 30, 3810-193 Aveiro, Portugal; 2 Department of Pathology, Portuguese Oncology Institute of Porto (IPO Porto), R. Dr. António Bernardino de Almeida, 4200-072 Porto, Portugal; 3 Cancer Biology and Epigenetics Group, IPO Porto Research Center (CI-IPOP), Portuguese Oncology Institute of Porto (IPO Porto)/Porto Comprehensive Cancer Centre (Porto.CCC) & RISE@CI-IPOP (Health Research Network), R. Dr. António Bernardino de Almeida, 4200-072 Porto, Portugal; 4 Department of Pathology and Molecular Immunology, School of Medicine & Biomedical Sciences, University of Porto (ICBAS-UP), Rua Jorge Viterbo Ferreira 228, 4050-513 Porto, Portugal

**Keywords:** unfolded protein response, breast cancer outcomes, therapy resistance, TCGA, immunohistochemistry, BiP/GRP-78

## Abstract

The endoplasmic reticulum chaperone BiP (also known as GRP-78 or HSPA5) maintains protein folding to allow cell proliferation and survival and has been implicated in carcinogenesis, tumor progression, and therapy resistance. BiP’s association with clinical factors and prognostic potential in breast cancer remains unclear. In this work, three types of analysis were conducted to improve the knowledge of BiP’s clinicopathological potential: (1) analysis of publicly available RNA-seq and proteomics datasets stratified as high and low quartiles; (2) a systematic review and meta-analysis of immunohistochemical detection of BIP; (3) confirmation of findings by BiP immunohistochemical detection in two luminal-like breast cancer small cohorts of paired samples (pre- vs. post-endocrine therapy, and primary pre- vs. metastasis post-endocrine therapy). The TCGA PanCancer dataset and CPTAC showed groups with high BiP mRNA and protein associated with HER2, basal-like subtypes, and higher immune scores. The meta-analysis of BiP immunohistochemistry disclosed an association between higher BiP positivity and reduced relapse-free survival. BiP immunohistochemistry confirmed increased BiP expression in metastasis, an association of BiP positivity with HER2 expression, and nuclear BiP localization with higher a tumor stage and poor outcome. Therefore, three independent approaches showed that BiP protein is associated with worse outcomes and holds prognostic potential for breast cancer.

## 1. Introduction

Breast cancer is a heterogeneous disease which represents the highest worldwide incidence of cancer in the female population. While prognosis is highly variable depending on the breast cancer subtype, breast cancer remains the leading cause of cancer death in women [1]. One of the current challenges is the identification of markers to improve stratification and predict responses to therapy. Several pre-clinical models have shown that breast cancer cells exploit the unfolded protein response (UPR) to survive therapy [2]. Independent studies have also shown an association of UPR proteins with breast cancer clinical factors, such as lymph node metastasis, estrogen receptor positivity, or decreased overall survival.

The UPR is activated in response to unfolded protein load in the endoplasmic reticulum and constitutes a cytoprotective stress response to maintain proteostasis and allow the adaptation of cells to environmental and metabolic changes [3,4]. The endoplasmic reticulum stressor sensor-binding immunoglobulin protein BiP, also known as GRP-78 or HSPA5, belongs to the HSP70 molecular chaperone family and resides primarily in the endoplasmic reticulum. This multifunctional protein targets misfolded proteins for proteasomal degradation, facilitates the folding and assembly of unfolded proteins to prevent intra and intermolecular aggregation, and controls the activation of transmembrane endoplasmic reticulum stress sensors (IRE1α, PERK and ATF6). Release of endoplasmic reticulum stress sensors from BiP leads to UPR activation and transcription of genes that promote survival and metabolic adaptation [5,6]. Expression of BiP is minimal in benign breast lesions, but it rises dramatically as breast cancer progresses [5,6,7,8]. HER2 amplification, acquired antiestrogen resistance and triple-negative tumors have been associated with increased BiP expression [9]. In addition, some publications correlated BiP protein overexpression with increased tumor size, increased tumor stage and grade, increased number of positive lymph nodes, distant metastasis, recurrent disease, and poor prognosis [5,10]. Furthermore, patients with positive BiP expression have lower overall survival and disease-free survival [11]. Recently, BiP was also found to be expressed on the cell surface of breast cancer cells, linked to early stages of the disease, high p53 and progesterone receptor (PR) levels, and a favorable prognosis in ER-positive tumors [12]. In triple-negative breast cancer, localization of BiP in the plasma membrane has been associated with increased apoptosis and tumor growth inhibition [13]. In addition, BiP controls calcium homeostasis and estrogen receptor alpha (ER)-mediated non-genomic signaling [4,14,15]. Due to its importance as a master regulator in the endoplasmic reticulum it is not surprising that BiP overexpression has been linked to breast cancer progression, as it allows proliferation, angiogenesis, and impacts the response to anticancer therapy [6,15,16,17,18,19,20].

Despite all the recent findings, to date, there is no evaluation of BiP association with clinical factors and pathways in large-scale datasets of mRNA and proteins such as TCGA [21] and CPTAC [22]. Additionally, there is no consensus on the correlation of BiP immunohistochemical expression with breast cancer clinical factors, and its marker potential is unknown. In this work, we carried out a systematic review of the literature and meta-analysis to investigate the associations between immunohistochemical detection of BiP in breast cancer and clinicopathological variables such as lymph node metastasis, molecular differentiation, tumor stage, grade and survival. Moreover, using the cBioPortal [23] to analyze the TCGA PanCancer dataset [21] and CPTAC [22], we were able to confirm that BiP mRNA and protein levels are associated with PAM50 breast cancer molecular subtype and the three immunohistochemical markers used in breast cancer diagnosis (ER, PR, and HER2), with them being higher in HER2+ and triple-negative breast cancer. This analysis also disclosed a potential association of BiP with the immune system, as well as identified pathways that explain these associations. Meta-analysis disclosed an association between higher BiP immunohistochemical positivity and relapse-free survival (RFS). Finally, using a small cohort of breast cancer cases, we were able to confirm increased BiP expression in metastatic tumors. BiP immunohistochemical positivity is associated with HER2 expression and its nuclear expression was related to a higher tumor stage and poorer outcome, highlighting the potential of this protein as a prognostic indicator for breast cancer.

## 2. Materials and Methods

### 2.1. Analysis of Public Datasets

The analysis of BiP mRNA expression in tumors, normal tissues, and metastasis was performed using RNA-seq or Gene-ChIP data available in the TNMplot [24] online tool assessed on 22 July 2022.

The publicly available database for tumor genomics and transcriptomics, the cBio Cancer Genomics Portal (cBioPortal) [23], accessed on 28 April 2022 [25], was used to perform an integrative analysis of treatment-naïve primary breast cancers. The TCGA PanCancer dataset, consisting of 1084 samples, of which only 10 samples had been treated [21], was stratified according to mRNA expression z-scores relative to all samples (log RNA Seq V2 RSEM) [BiP-Low (BiP-L) (−2.37–0.66; *n* = 270 samples)) and BiP-High (BiP-H) (0.58–5.05; *n* = 271 samples)]. The same procedure was applied to the proteogenomic landscape of breast cancer (CPTAC, Cell 2020) dataset [BiP-L (−1.68–−0.48; *n* = 30 samples) and BiP-H (0.48–3.16; *n* = 31 samples)]. CPTAC consists of proteomics data of 122 treatment-naïve primary breast cancer samples analyzed by mass spectrometry and the corresponding clinical data [26]. These two datasets were used to analyze the associations of BiP mRNA or protein levels with clinicopathological factors, survival outcomes, and pathway overrepresentation. Differences were considered statistically significant if *p*val and *q*Val ≤ 0.05. Differential expression of gene or protein levels was considered significant if Log FC ≥ 0,4 and *p* > 0.05. Pathway enrichment was carried out using values/ranks for each differentially expressed gene or protein, and String V11.5 [27,28] and both Kegg and Reactome Pathways were analyzed and considered significantly enriched if the false discovery rate (FDR) ≤ 0.05.

Additionally, the TCGA-BRCA raw counts and FPKM data were downloaded on 20 March 2022 from NCI Genomic Data Commons (GDC) using the TCGAbiolinks package (version 2.22.4, Bioconductor, Seatle, IL, USA)), United States [29] in R (version 4.1.2) and they were used to compare BiP mRNA expression across breast cancer molecular subtypes and identify correlations between BiP expression and immune and other stromal signatures. To compare BiP mRNA expression across breast cancer molecular subtypes, raw counts were normalized using the cumulative sum scaling (CSS) method from the metagenomeSeq package, Bioconductor [30]. The BiP normalized raw counts were plotted against the different breast cancer subtypes and compared by an ANOVA test followed by a Tukey’s test.

To identify correlations between BiP expression and immune and other stromal signatures, BiP was first defined as highly or lowly expressed based on upper and lower quartiles of the raw counts, respectively. The samples corresponding to the middle quartiles were considered unchanged and therefore removed. Genes with less than 1 FPKM in both high and low BiP patients were considered not expressed and removed. Genes that were not present in at least a quarter of the samples were also filtered out, and this was completed based on counts per million using the edgeR package (version 3.36.0, Bioconductor) [31,32,33]. The raw counts for the remaining samples were used to identify the infiltrating immune and stromal scores for samples expressing high and low BiP using the immunedeconv package (version 2.0.4, Omnideconv, Innsbruck, Austria) [34].

### 2.2. Meta-Analysis of BiP Immunohistochemistry in Breast Cancer Samples

This study was submitted to PROSPERO on 28 April and registered on 10 May 2022 (CRD42022328977).

#### 2.2.1. Search Strategy

To identify reports that performed immunohistochemical analysis of BiP in breast cancer, free text words, as well as singular and plural forms of the key terms, were used in the queries. The key words were searched for in the title and abstract to identify all potentially relevant articles in PubMed/Medline and Scopus databases. In PubMed/Medline, the query used was: ((mammary cancer[Title/Abstract]) OR (mammary carcinoma[Title/Abstract]) OR (mammary tumor[Title/Abstract]) OR (mammary neoplasms[Title/Abstract]) OR (breast cancer[Title/Abstract]) OR (breast carcinoma[Title/Abstract]) OR (breast tumor[Title/Abstract]) OR (breast neoplasms[Title/Abstract])) AND ((HSPA5[Title/Abstract]) OR (Endoplasmic reticulum chaperone BiP) OR (GRP-78) OR (GRP78) OR (BiP)). In Scopus, the query was: TITLE-ABS-KEY ((“mammary cancer”) OR (“mammary carcinoma”) OR (“mammary tumor”) OR (“mammary neoplasms”) OR (“breast cancer”) OR (“breast carcinoma”) OR (“breast tumor”) OR (“breast neoplasms”)) AND ((hspa5) OR (“endoplasmic reticulum chaperone BiP”) OR (grp-78) OR (grp78) OR (bip)) AND (LIMIT-TO (DOCTYPE, “ar”). The search was unlimited for articles published up to September 2021. Existing reviews and reference lists were hand searched for studies missed by the initial query. The search was updated on the 8 July 2022 in both databases, but no new studies were identified.

#### 2.2.2. Eligibility and Data Collection

Two of the authors assessed all the retrieved references for eligibility based on the information provided in the title and abstract. All the potentially eligible full-text articles were retrieved for full-text analysis using the inclusion criteria: (1) immunohistochemical analysis of BiP expression levels in human breast cancer samples; (2) studies with sufficient data to evaluate methodological quality and to perform a meta-analysis, which includes a clear description of the study population and immunohistochemical methods (i.e., tissue treatment, antibodies used, positive controls, and cut-off used to assign expression status); (3) correlation between BiP expression and clinicopathological parameters discussed; (4) more than 5 samples analyzed; (5) when different papers reported BiP expression from the same patient cohort, only the most recent or the most complete study was included. Only original reports were considered. Letters, reviews, case reports, editorials, and comments were excluded. Selected references for which a full-text article was not available after contact with dedicated libraries and/or with corresponding authors were also excluded (Supplementary Table S1).

#### 2.2.3. Data Analysis

The analysis was performed in R (version 4.1.2, R Core Team, Vienna, Austria) and the packages meta, dmetar for statistics and netmeta for network meta-analysis [35,36]. The prevalence, Cohen’s d, and relative risk were calculated as point estimates of the association between the expression of BiP and the patients’ clinicopathological characteristics. Pooled prevalence indicates the proportion of positive staining for each marker. Pooled relative risk was calculated for differences in BiP positivity regarding recurrence-free survival. Between-studies, heterogeneity was estimated using heterogeneity index (I2) statistics [37]. In case of substantial heterogeneity between the studies (I2 > 50%), only the results from a random-effects model were considered for further analysis; otherwise, a fixed effect model was used for the pooled statistical analysis, and a meta-regression analysis (mixed-effects model) was performed using an ‘adjusted effect’ to potential moderators. All of the results were considered statistically significant if *p* < 0.05. Sensitivity analysis was carried out to assess the robustness of the results by removing individual studies from the meta-analysis and assessing the effect on the pooled results. The publication bias was evaluated using funnel plots and two-sided Egger’s tests. Subgroup meta-analyses were performed to explore sources of heterogeneity for the sample type. Meta-regression was also used to assess the influence of the following factors in the BiP proportion of positive cases: (1) sample type, (2) menopausal status, (3) tumor stage, (4) lymph node metastases, (5) tumor grade, (6) ER-positivity, (7) HER2-positivity, and (8) age.

### 2.3. Immunohistochemical Detection of BiP

#### 2.3.1. Patient Sample Collection and Characterization

The clinicopathological data of the patient cohorts are presented in Supplementary Tables S2 and S3. Tumor samples consisted of formalin-fixed paraffin-embedded tumor tissues from breast cancer patients. The slides were reviewed by the pathologist involved in the study (JL). Staging was performed according to the most recent AJCC staging manual and the histological subtypes considered in the latest World Health Organization classification. Molecular subtype was determined by immunohistochemistry, with ER, PR, HER2 (with confirmation by FISH in case of a 2+ immunohistochemical result), and Ki67, as recommended by the College of American Pathologists. All of the patients were treated by the same multidisciplinary team. The samples used in this study were obtained from the archive of the Department of Pathology of the Portuguese Institute of Oncology of Porto (IPO-P). Their use was approved by IPO-P’s Ethical Committee (CES IPO: 369/2017).

#### 2.3.2. Immunohistochemistry

Immunohistochemical staining was performed on breast cancer tissue samples using the anti-BiP/GRP78 antibody (HPA038845, 1:400, Sigma-Aldrich, St. Louis, MO, USA) validated by the Human Protein Atlas as specific for this protein [38] or anti-Ki-67 (Ab15580, 1:250, Abcam, Cambridge, United Kingdom). Briefly, 4 μm-thick sections were deparaffinized in xylene and hydrated in graded alcohols. Endogenous peroxidase activity was blocked with a 3% hydrogen peroxide solution. For antigen retrieval, sections were immersed in citrate buffer pH = 6.0 (C_6_H_5_NaO_7_.2H_2_O 2.94 g/L) and heated in a microwave oven for 20 min. The slides were permeabilized with 0.25% Triton X-100 and blocked with 0.2% bovine serum albumin in phosphate buffer saline (PBS), followed by incubation with the primary antibody overnight in a humidified chamber. The slides were washed and the secondary biotinylated antibody (SAB3700856, Sigma, 1:400) was incubated for 2 h at room temperature. The slides were washed with PBS and then incubated with freshly prepared VECTASTAIN ABC Reagent (Vector Laboratories, Burlingame, CA, USA) for 30 min. The slides were developed with 3,3-diaminobenzidine substrate-chromogen solution and subjected to Mayer hematoxylin counterstaining. The immunohistochemical slides were evaluated blinded to patient identity. The number of positive stained cells and total cells were enumerated and the % of positive stained cells was obtained. For BiP, the scoring system combined staining intensity (0, no staining; 1, weak; 2, moderate; 3, strong) and % of stained cells (0, no staining; 1, 1 to <10%; 2, 10 to <50%; 3, 50 to 100%). The final immunohistochemical score was determined by multiplying the individual scores of the previous variables and considered positive if ≥2 (in accordance with most of the studies included in the meta-analysis which used the same cut-off). To evaluate BiP and Ki67 index, the number of positive and total cells were counted, and the % of positive cells was determined. Nikon eclipse Ti-U was used to conduct bright-field microscopy and the slides were quantified in three random field pictures at 200× magnification.

#### 2.3.3. Statistical Analysis

Statistical analyses were performed using the software SPSS version 28.0.1.1, IBM, Armonk, NY, USA and GraphPad Prism version 6.0, Dotmatics, San Diego, CA, USA. Wilcoxon tests were used for analysis of human breast cancer paired tissue samples. The relationships between BiP immunohistochemical staining and clinicopathologic variables were analyzed using the Fisher’s exact probability test. The associations between the expression levels of BiP and Ki67 were analyzed by the Spearman’s rank correlation. Differences were considered significant if *p* < 0.05.

## 3. Results

### 3.1. Stratification of Public Datasets by BiP Differential Expression Correlates with Breast Cancer Molecular Subtype and Immune Score

**Figure 1:**
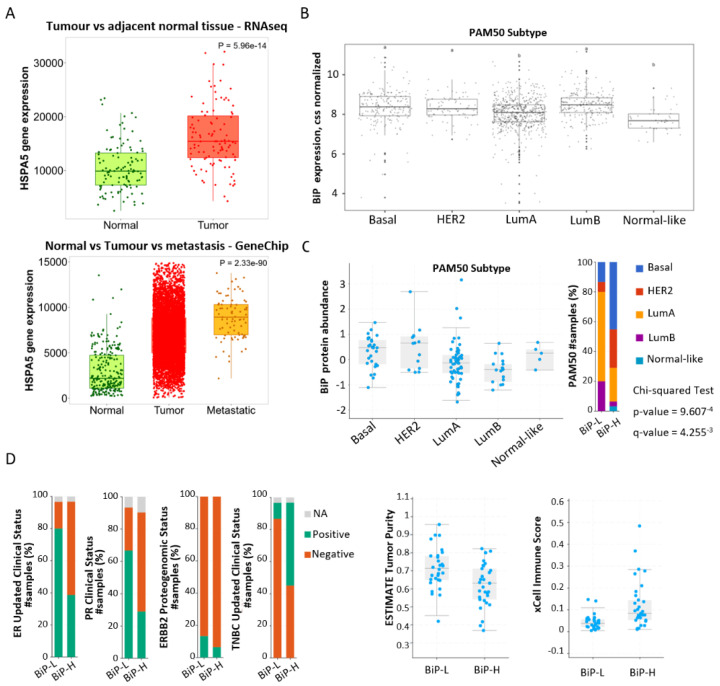
Association of BiP differential expression with breast cancer clinical factors and pathways. (**A**) Analysis of BiP mRNA expression in breast tumor tissue compared to normal and metastatic tissues using gene-chip- or RNA-seq-based data available in TNMplot; (**B**) Analysis of BiP mRNA expression across PAM50 subtypes using TCGA-BRCA data in R. ANOVA followed by Tukey’s test: significant difference at 5% level of significance. (**C**) Analysis of BiP protein levels across breast cancer subtypes using the CPTAC dataset in cBioPortal. (**D**) BiP correlation with clinical factors. BiP protein abundance in the CPTAC dataset was used to stratify the BiP higher and lower quartiles (BiP-H and BiP-L) and an analysis carried out using cBiopPotal. ER—estrogen receptor, PR—progesterone receptor, TNBC—triple-negative breast cancer, and ERBB2—receptor tyrosine-protein kinase erbB-2.

The analysis of BiP expression in normal and cancer tissues showed that BiP mRNA levels are higher in breast cancer tissues vs. normal tissues [non-paired, *p* = 9.13 × 10^−84^ or paired adjacent tissue, *p* = 5.96 × 10^−14^] and highest in the metastases (non-paired tissues, *p* = 2.33 × 10^−90^; Figure 1A). In the TCGA BRCA dataset, BiP mRNA was lowest in luminal A and normal-like subtypes as compared with basal, HER2 and luminal B subtypes which did not vary with each other (Figure 1B). Analysis of PAM50 breast cancer molecular subtype representation in the CPTAC dataset confirmed higher basal and HER2 subtypes in the high BiP (BiP-H) group and showed higher BiP protein abundance in these subtypes. The protein abundance ratio was lower in luminal A and B, resulting in a higher number of cases of these two subgroups in the low BiP (BiP-L) group (Figure 1C).

**Figure 2:**
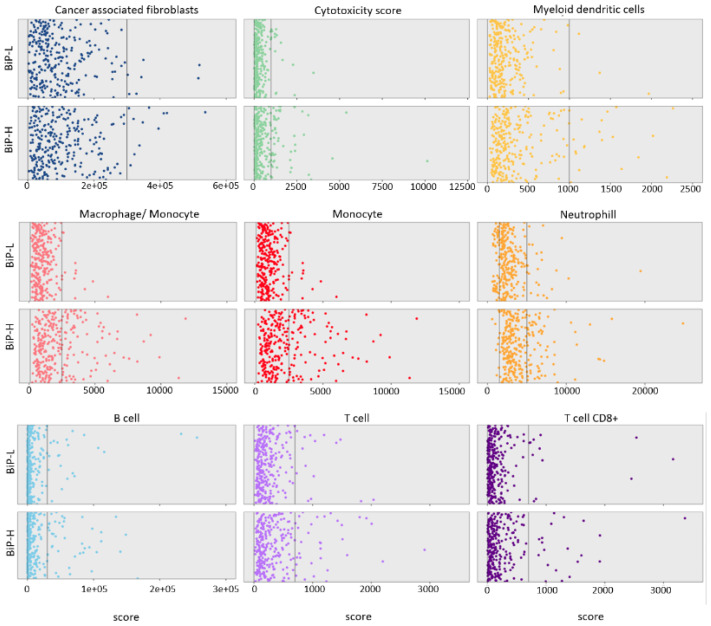
Association of BiP differential expression (high vs. low; BiP-H and BiP-L, respectively) with tissue-infiltrating immune and stromal cell populations using the mcp_count method from the immunodeconv package and TCGA-BRCA data overall.

Based on protein abundance ratio (CPTAC dataset), the BiP-L group was significantly correlated with ER, PR, and ERBB2 positivity and with a higher estimate tumor purity score (Figure 1D and Table 1). On the other hand, the BiP-H group was significantly correlated with triple-negative breast cancer, TOP2A proteogenomic status, and the xCell immune score (Figure 1D and Table 1). To identify the stromal cell types that could be contributing to these correlations, we used the mcp_count method [39] from the immunodeconv package [34] on the TCGA BRCA RNA-seq dataset. An enrichment of macrophages and monocytes together with a slightly higher increase in neutrophiles and B cells was identified in the BiP-H group (Figure 2). There was no significant difference in overall survival, disease-free survival, or progression free survival between the BiP-H group and the BiP-L group.

In BiP-L vs. BiP-H TCGA PanCancer Atlas groups, the pathways overrepresented where all in the direction of the BiP-H group with the top ten Kegg pathways including protein processing in the endoplasmic reticulum (FDR = 3.47 × 10^−12^), the innate immune, and the adaptive immune system (FDR = 2.16 × 10^−24^ and FDR = 2.22 × 10^−14^, respectively; Table 2).

As expected, UPR (FDR = 8.16 × 10^−7^), IRE1alpha activates chaperones (FDR = 1.24 × 10^−5^), and XBP1(S) activate chaperone genes (FDR = 1.79 × 10^−5^), as well as pathways regulating proteostasis such as lysosome (FDR = 0.0031) and proteasome (FDR = 0.00023) were amongst the significantly overrepresented pathways in BiP-H. In the CPTAC dataset, the top ten enriched pathways in the BiP-H group were protein processing in the endoplasmic reticulum (FDR = 9.15 × 10^−6^), the innate immune system (FDR = 3.49 × 10^−8^), and UPR (FDR = 0.0077); while in the BiP-L group, DNA repair (FDR = 0.00099) and estrogen-dependent gene expression (FDR = 0.0020) stood out as the most relevant cancer-related pathways (Table 3).

In summary, BiP mRNA and protein levels are significantly associated with PAM50 breast cancer molecular subtype and the three immunohistochemical markers used in breast cancer diagnosis (ER, PR, and HER2). Higher expression is observed in HER2+ and triple-negative breast cancer. Stratifying breast cancer samples by BiP mRNA and protein enriched for known functional effects of BiP on proteostasis including activation of the IRE1alpha/XBP-1 UPR arm. Additionally, stratification according to BiP also disclosed a potential association with the microenvironment innate immune system (macrophages, monocytes and neutrophiles) as well as adaptive immune system (B cells) pathways.

### 3.2. A Meta-Analysis of BiP Immunohistochemistry Identifies An Association with A Higher Risk of Recurrence

**Figure 3:**
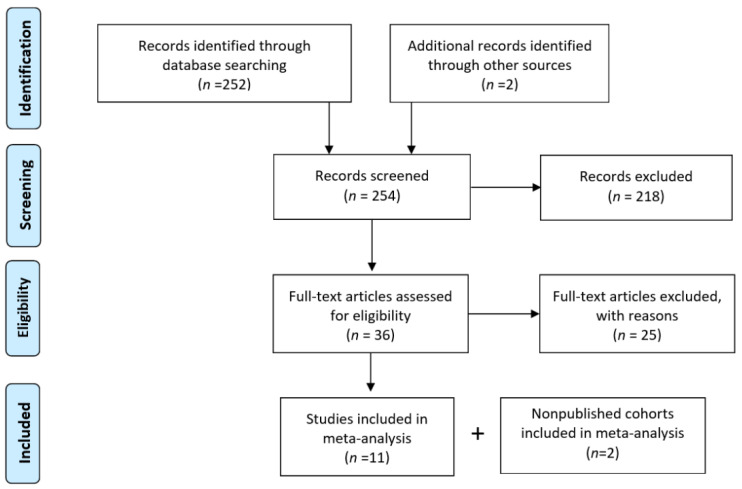
Flowchart summarizing the literature selection process for the systematic literature revision and meta-analysis of BiP immunohistochemistry in breast cancer.

**Figure 4:**
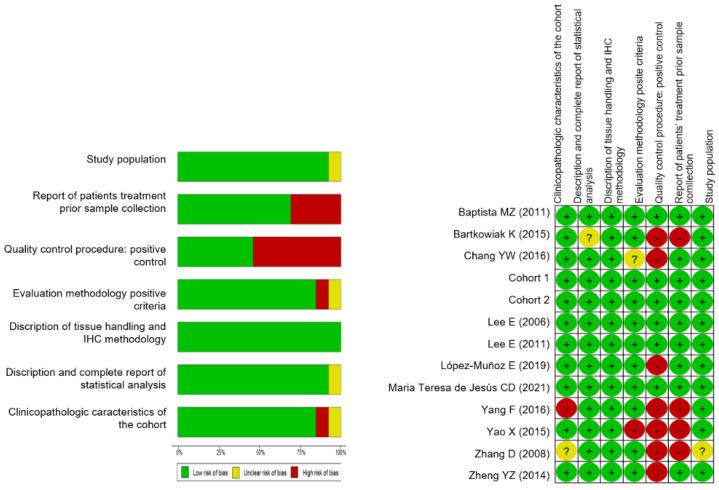
Risk of bias assessment per criteria presented as percentages across all included studies and risk of bias in individual studies: Baptista, M.Z. (2011) [11], Bartkowiak, K. (2015) [42], Chang, Y.W. (2016) [43], Cohort 1, Cohort 2, Lee, E. (2006) [41], Lee, E. (2011) [48], López-Muñoz, E. (2019) [47], Maria Teresa de Jesús, C.D. (2021) [46], Yang, F. (2016) [44], Yao, X. (2015) [5], Zhang, D. (2008) [45], Zheng, Y.Z. (2014) [10]. + (green circles): low risk; ? (unclear, yellow circles): unclear risk; - (red circles): high risk.

The results from the analysis of public datasets prompted us to investigate the potential of BiP immunohistochemical detection in breast cancer diagnosis and prognosis. We carried out a systematic revision and meta-analysis including retrospective published studies from 2006–2022. Eleven studies were eligible for the analysis (Table 4 and Figure 3) with a total of 1081 breast cancer samples (48–213 samples per study). Additionally, we analyzed, by immunohistochemistry, two cohorts from IPO-P (cohort 1 and 2 untreated luminal tumors; Table 5) totalizing 1107 tumor samples. The REMARK risk of bias [40] showed that a lack of information about quality control/the inclusion of positive control in the study was the most common factor contributing to the bias (high risk in 50% of the studies; Figure 4), followed by insufficient information about patient treatment prior to sample collection (with nearly 50% of the studies not reporting). Poor or insufficient description of the clinicopathological characteristics of the cohorts was also identified in 17% of studies (Figure 4).

**Table 4:** Characteristics of the studies included for meta-analysis of GRP78/BiP in breast cancer.

Study	N	Positive Cases	Antibody	Histology	Sample	Cutoff	Pre/Post Menopause	Low/High Stage	Low/ High Grade	Lymph Nodes +/ Lymph Nodes −	LVC Invasion/ No LVC Invasion	ER+/ ER−	HER2+/ HER2−
Baptista, M.Z. (2011) [6]	106	93	C50B12	N/A	TMA	score	68/38	25/81	20/86	93/13	33/73	76/30	27/79
Zheng, Y.Z. (2014) [10]	213	112	11587-1-AP	IDC	TMA	score	98/115	N/A	123/55	83/130	N/A	90/123	83/130
Lee, E. (2006) [41]	127	85	sc-13968	IDC + ILC + other	Tissue	score	66/61	116/11	52/52	106/21	50/77	97/27	23/76
Bartkowiak, K. (2015) [42]	182	161	C50B12	IDC + ILC + other	TMA	score	N/A	167/14	102/73	67/114	15/131	124/37	6/157
Chang, Y.W. (2016) [43]	108	60	sc-13968	IDC	Tissue	score	N/A	N/A	N/A	N/A	N/A	N/A	N/A
Yao, X. (2015) [5]	104	68	sc-1051	IDC + other	Tissue	score	66/38	70/34	44/60	70/34	N/A	69/35	31/73
Yang, F. (2016) [44]	50	49	sc-13968	N/A	Tissue	score	N/A	N/A	N/A	N/A	N/A	N/A	N/A
Zhang, D. (2008) [45]	80	50	610979	N/A	TMA	score	N/A	N/A	6/8	8/6	N/A	6/8	32/48
María Teresa de Jesús, C.D. (2021) [46]	48	35	ab21685	IDC + other	TMA	score	24/31	N/A	N/A	N/A	N/A	27/26	13/40
López-Muñoz, E. (2019) [47]	15	14	ab21685	IDC	Tissue	score	N/A	11/4	N/A	11/4	N/A	11/4	1/13
Lee, E. (2011) [48]	48	29	sc-13968	IDC + other	Tissue	score	30/18	N/A	17/30	28/12	N/A	N/A	21/25

N/A—not available; IDC—invasive ductal carcinoma; ILC—invasive lobular carcinoma; TMA—tissue microarray; LVC—lymphovascular.

**Table 5:** Characteristics of the IPO-P’s cohort samples included in the meta-analysis of BiP/GRP78 in breast cancer.

Study	N	Positive Cases	Antibody	Histology	Sample	Cutoff Criteria	Low Stage/ High Stage	Low Grade/ High Grade	Lnodes/ No Lnodes	ER+/ ER−	HER2+/ HER2−
cohort 1	14	11	HPA038845	IDC + ILC + other	Tissue	score	9/5	11/3	1/13	14/0	2/12
cohort 2	12	9	HPA038845	IDC + ILC + other	Tissue	score	7/5	5/7	12/0	12/0	3/9

IDC—invasive ductal carcinoma; ILC—invasive lobular carcinoma.

**Figure 5:**
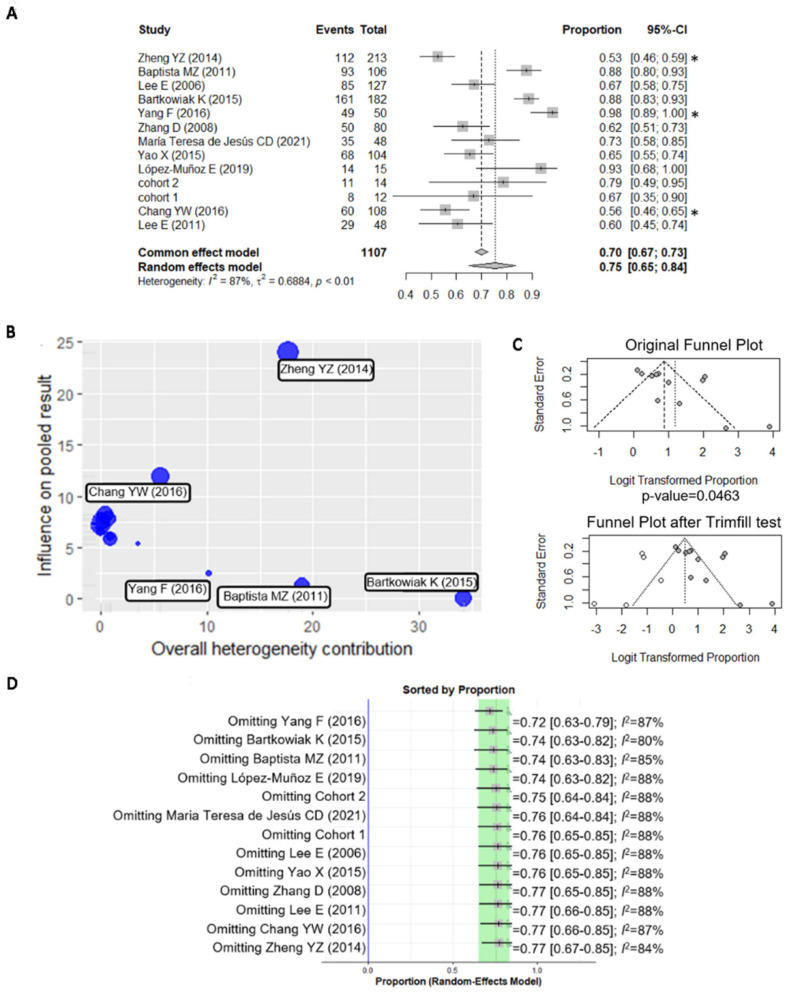
Quality assessment of the meta-analysis. (**A**) Forest plot showing the pooled results of BiP positive expression in breast cancer samples in the included studies: Baptista, M.Z. (2011) [6], Bartkowiak, K. (2015) [42], Chang, Y.W. (2016) [43], Lee, E. (2006) [41], Lee, E. (2011) [48], López-Muñoz, E. (2019) [47], Maria Teresa de Jesús, C.D. (2021) [46], Yang, F. (2016) [44], Yao, X. (2015) [5], Zhang, D. (2008) [45], Zheng, Y.Z. (2014) [10], cohort 1 and cohort 2; * identifies the possible outliers. CI—confidence interval. (**B**) Baujat plot comparing the weight of each study to the overall BiP staining heterogeneity; (**C**) funnel plot showing the asymmetry and publication bias for BiP and the addition of five studies with the trim-fill test. (**D**) Sensitivity analysis showing that omitting one study at a time does not influence the overall results.

Considering all of the studies and the IPO-P cohorts, 775 samples (70%) were positive for BiP, with a positive % range of 53–98% (Figure 5A). The sensitivity analysis identified the studies by Zheng, Y.Z. (2014) [10], Yang, F. (2016) [44], and Chang, Y.W. (2016) [43] as outliers of the pooled results (Figure 5B). The study from Bartkowiak, K. (2015) [42] had the highest contribution to the overall heterogeneity (I2 = 88%) and the study by Zheng, Y.Z. (2014) [10] had the highest influence on the pooled results. The publication bias was evaluated using funnel plots and two-sided Egger’s tests (Figure 5C). Omitting one study at a time did not influence the overall results; therefore, all of the studies were maintained for the subsequent analyses (Figure 5D).

Meta-regression was used to assess whether the heterogeneity between the studies could be explained by clinicopathological variables (Table 6). In the mixed-effects model, grade (*p* = 0.0498), ER expression (*p* = 0.0371), HER2 expression (*p* < 0.0001), and age (*p* = 0.0018) were unveiled as significant sources of variation that influence BiP positivity. These results agree with the association of BiP protein levels with ER and the high levels in HER2 molecular subtypes (Figure 1D,C).

**Figure 6:**
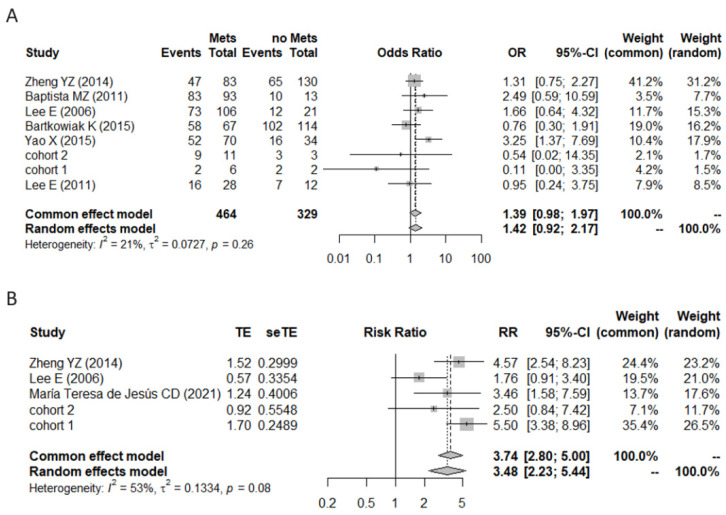
Forest plots for the binary meta-analysis. (**A**) Association between BiP positivity and lymph node metastasis in the studies Baptista, M.Z. (2011) [6], Bartkowiak, K. (2015) [42], Lee, E. (2006) [41], Lee, E. (2011) [48], Yao, X. (2015) [5], Zheng, Y.Z. (2014) [10], cohort 1 and cohort 2.; (**B**) Association between BiP positivity and recurrence-free survival [RFS] in the studies Lee, E. (2006) [41], Maria Teresa de Jesús, C.D. (2021) [46], Zheng, Y.Z. (2014) [10], cohort 1 and cohort 2. Individual study estimates of crude odds ratios and 95% confidence intervals (CI). Due to heterogeneity (I2), only random effects estimates were considered. Error bars indicate confidence intervals.

A binary meta-analysis was performed to determine the relationship between BiP positivity and tumor grade, tumor stage, lymph node metastasis, menopausal status, ER expression, and HER2 expression. Although no significant association was found between lymph node metastasis and BiP positivity (*n* = 793, from 8 studies; I2 = 21%, CI = [0.92–2.17]) the analysis revealed a tendency for higher BiP immunodetection in metastatic tumors (post-treatment A), which agrees with BiP mRNA levels being highest in the metastasis tissues vs. primary tissues (Figure 1A). Contrary to the expected results, no significant differences were detected between BiP positivity and tumor stage (*n* = 543, from 6 studies, I2 = 62%, CI = [0.30–2.47]), tumor grade (*n* = 692, from seven studies, I2 = 28%, CI = [0.47–1.31]), menopausal status (*n* = 598, from five studies, I2 = 26%, CI = [0.75–1.85]), ER expression (*n* = 734, from seven studies, I2 = 0%, CI = [0.62–1.33]), or HER2 expression (*n* = 837, from nine studies, I2 = 13%, CI = [0.58–1.42]). However, BiP positivity was significantly associated with a higher risk of recurrence (*n* = 414, from five studies, I2 = 53%, RR = 3.48, *p*-value = 0.08 CI = [2.23–5.44]; Figure 6B). This conclusion should be carefully considered due to the low number of studies analyzed and the lack of correction for co-funding factors; still, the pooled effect was strong and reflected the results of the individual studies.

In summary, there are currently few studies to confidently associate BiP immunohistochemical positivity with clinical factors. Still, in line with the results from mRNA and protein public datasets, our data clearly show that the breast cancer diagnostic markers ER and HER2, as well as tumor grade, influence the heterogeneity of results. In addition, aligned with public dataset analysis, higher BiP immunohistochemical positivity was associated with recurrence-free survival and was borderline with metastasis, suggesting BiP as a prognostic indicator.

### 3.3. Effect of Therapy and Metastasis on BiP Expression in Breast Cancer

The analysis of CPTAC showed that BiP protein is lowest in the luminal breast cancer molecular subtypes as well as in tumors expressing ER, and, logically, the BiP-L group had significant overrepresentation of the ER signaling pathway (Table 3). Still, luminal tumors express BiP, which preclinical studies have shown can induce ER non-genomic signaling and interact at the protein level with ERα^2^. Therefore, we aimed to investigate how the detection of BiP by immunohistochemistry in luminal tumors is influenced by antiestrogen therapy and metastasis.

Immunohistochemical staining of BiP was performed in 52 breast cancer cases retrieved from IPOP’s biobank. Cohort 1 included 14 biopsies from ER+/HER2+ or −, luminal B, or luminal-like primary breast tumors that are treatment-naïve and the paired tumor samples after neoadjuvant antiestrogen treatment. Tumors with ductal, lobular, mixed, or neuroendocrine histological differentiation, staged from IIB to IV, were included. In total, 28 samples from patients with ages from 40 to 87 years old were analyzed in cohort 1. Cohort 2 included 12 ER+/HER2 + or −, luminal or luminal-like, treatment-naïve primary breast tumors and paired metastasis arising after endocrine treatment. Ductal, lobular, or mixed histological types were included as well as tumors staged between I and III. A total of 24 samples from patients with ages ranging 30 to 69 years-old were analyzed. The detailed clinicopathological characteristics of the two cohorts are presented in Supplementary Tables S2 and S3, respectively.

**Figure 7:**
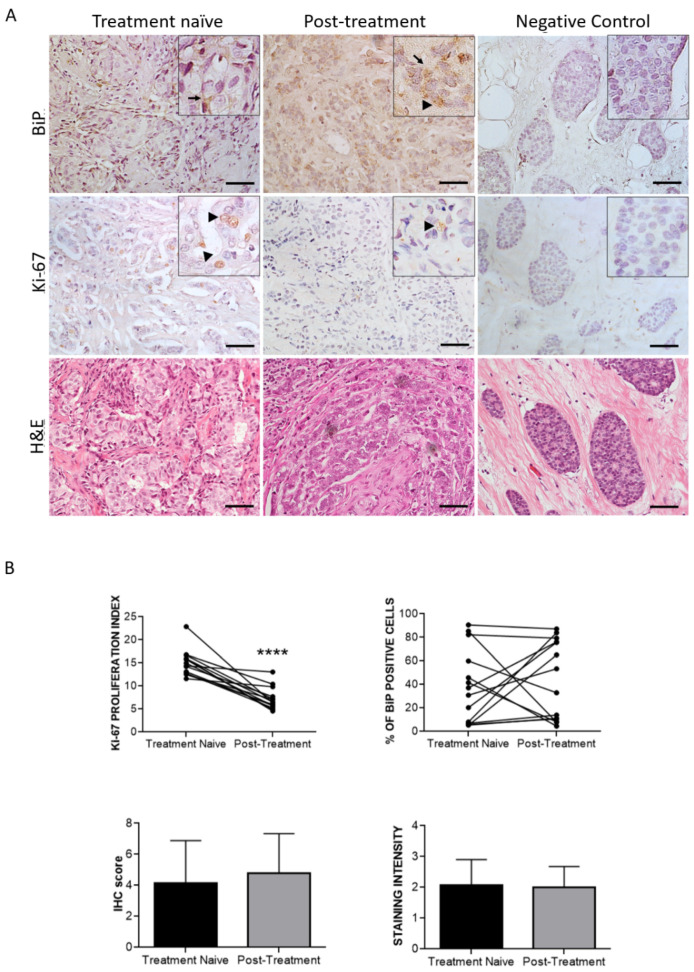
Representative pictures of BiP and Ki67 immunostaining and respective H&E in treatment-nave biopsies vs. their matched post-treatment tumors. (**A**) Arrows indicate cytosolic BiP expression while arrowheads show BiP and Ki67 nuclear localization in neoplastic cells. All pictures were from the same invasive ductal carcinoma case as seen in the corresponding H&E slide showing irregular and angulated tubules infiltrating the breast stroma. Scale bars: 50 μm (**B**) Graphs comparing % Ki67 index and %BiP positive cells, as well as BiP immunohistochemical (IHC) score in paired treatment-naïve biopsies and post-treatment tumors (*n* = 14). ****: *p* < 0.0001; Wilcoxon paired test.

In cohort 1, all except two patients responded well to antiestrogen therapy as shown by a reduction of Ki67 index (Figure 7). In treatment-naïve tumors, BiP positive staining was detected in 78.5% (11/14) of the samples, with them being predominantly cytosolic (72.4%) (Figure 6A) and was significantly correlated with HER2 expression (*p*-value = 0.046) and tumor grade (*p* = 0.014) (Table 7).

After antiestrogen treatment, BiP positivity was detected in 85.7% (12/14) of the samples, but this increase was not significant. Interestingly, the subcellular localization of BiP was altered by treatment, with it being both cytosolic and nuclear in eight samples, a 57.1% increase in the nuclear + cytosolic staining compared with treatment-naïve tumors, although the percentage of positive cells and staining intensity remained unchanged (Figure 7B). BiP nuclear expression after antiestrogen treatment was found to be significantly associated with patient death (*p*-value = 0.043) and with higher tumor stage (*p*-value = 0.038) (Table 8).

**Figure 8:**
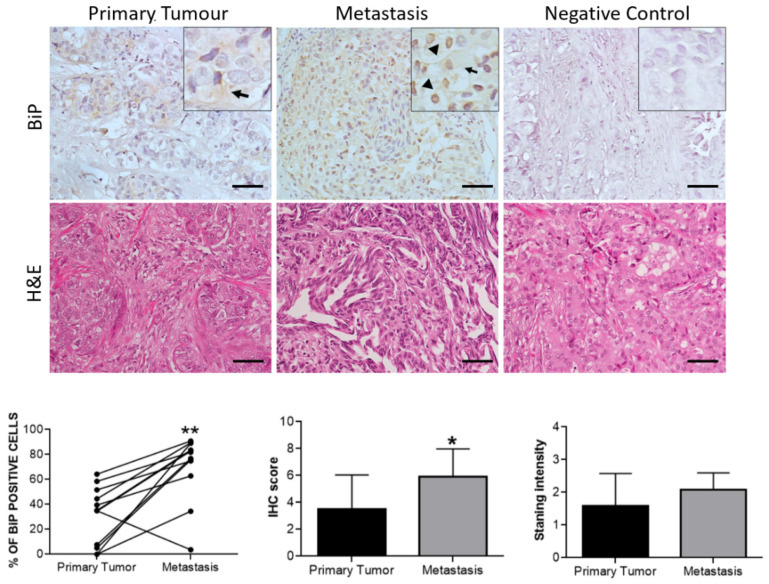
Representative pictures of BiP immunostaining and respective H&E in treatment-naïve primary tumors vs. their paired metastasis. Arrows indicate cytosolic BiP expression while arrowheads show BiP nuclear localization in neoplastic cells. All pictures were from the same invasive ductal carcinoma case, as seen in the corresponding H&E slide showing irregular ducts infiltrating the breast stroma. Scale bars: 50 μm. The bar graphs comparing %BiP positive cells, BiP IHC score, and BiP IHC intensity of staining in paired treatment untreated tumors vs. post-treatment metastases (*n* = 12). *: *p* < 0.05, **: *p* < 0.01; Wilcoxon paired test.

In cohort 2, BiP positivity was detected in 66.7% (8/12) of the primary tumors and in 91.7% (11/12) of their paired metastasis, with the percentage of positive cells significantly being increased in the latter (Figure 8). The subcellular localization of BiP in the metastases was nuclear and cytosolic in 83.3% of the samples, representing a 33.3% increase compared with the paired primary tumors (Figure 8). No significant associations were found between BiP immunodetection and clinicopathological characteristics in this cohort (Table 9 and Table 10).

In summary, despite the small size of the cohorts analyzed, BiP immunohistochemical positivity associated with HER2 expression which is in line with the results from the meta-analysis, mRNA datasets, and protein public datasets. Equally aligned with previous results, BiP expression was found to be higher in metastatic tumors, with its nuclear expression being associated with higher a tumor stage and poorer outcome, further suggesting, that BiP could be a prognostic indicator for breast cancer.

## 4. Discussion

BiP/GRP78 in the inactive form acts as a key repressor of UPR, but once activated by binding to unfolded proteins, it plays a key role in protein folding and quality control in the endoplasmic reticulum lumen [49]. Cancer progression is associated with genomic instability, hypoxia, and oxidative stress, which all known to increase the dependency on adaptive stress responses that protect from proteotoxic stress related to the accumulation of misfolded proteins. Breast cancer cells usually overexpress molecular chaperones, including BiP, which facilitate the pro-survival and cytoprotective response of cancer cells to environmental stress [8]. Analyzing public datasets, immunohistochemical studies, and our own cohorts, we showed that BiP protein expression is associated with prognosis clinicopathological factors and further validation should establish its potential as an immunohistochemical marker to be used in breast cancer diagnostics.

The analysis of RNA-seq data confirmed previous studies showing higher BiP expression in breast cancer tissues vs. normal tissues and the further BiP increase in metastatic tumors [5,7,42,50]. These findings are in agreement with the meta-analysis and our own cohort 2. This confirms previous pre-clinical data showing that BiP knockdown results in decreased breast cancer cell invasion, proliferation, and metastasis [43,51,52]. Moreover, even though there was no association with tumor stage, the meta-analysis and our own cohorts also showed a significant association between increased BiP positivity and tumor stage. This may be explained by the higher BiP positivity in tumors expressing HER2, which tend to be more aggressive.

When BiP protein was stratified according to the lowest and highest quartile using the CPTAC dataset, a remarkable increase in the number of ER- and PR-positive tumors was significantly represented in the tumors with lowest BiP levels, which was also in agreement with mRNA expression showing significantly lower BiP in luminal A breast cancer vs. the other molecular subtypes. The higher number of ERBB2-positive tumors in lowest BiP group is puzzling given the strong correlations found between BiP and the HER2+ subtype (TCGA, CPTAC, and immunohistochemistry), and this may be due to ERBB2 expression in luminal B molecular subtype. In addition, it is already documented that induction of BiP at protein level is variable and does not always correspond with the transcript level [18], thus these associations deserve further attention.

Higher expression of BiP protein significantly correlated with triple-negative breast cancer and the TOP2A proteogenomic status, with poor differentiation (basal). This together with its positive association with higher grade and metastasis clearly signals BiP as a poor prognosis marker. Additionally, using Immunodeconv version 2.0.4 [34], we observed neutrophils, macrophages, and monocyte signatures in the BiP-high tumors. Although these cells need to be confirmed in the tumor tissue slides, increasing evidence points toward neutrophils and a higher neutrophils/lymphocyte ratio as markers of disease aggressiveness in ER- and HER2-negative breast cancers [53] as well as in triple-negative breast cancers [54] and response to chemotherapy [55], endocrine therapy [56] or trastuzumab treatment [57]. On the other hand, monocytes and macrophages have both pro- and anti-tumor effects in the breast cancer microenvironment [58] and it will be interesting to design future studies to evaluate the correlation of both phenotypes with BiP.

One of the major limitations of this meta-analysis is the high degree of heterogeneity and the potential confounding effects that remain undisclosed. The main methodological discrepancies found between the studies relate to the different antibodies used. Indeed, the antibodies are a significant source of variability as, despite being validated for immunohistochemistry and clinical application, they may yield dissimilar results. In addition, different scoring systems may also explain some heterogeneity since the studies with the higher proportion of BiP-positive cases are the ones that stablished a 1% cut-off [6,42,44]. Even though the sample size was sufficient to disclose a statistically significant prognostic value for BiP, due to the limited statistical power, the sample size should be expanded.

One result that stood out from the meta-analysis showed that BiP positivity was associated with a higher risk of recurrence, although only three studies [10,41,46] the besides IPO-P cohorts were included in this analysis (note that the study by Lee et al., 2011 [48], was not considered since RFS is calculated in a specific subpopulation of breast cancer patients treated with taxanes). While this is supported by the significant association with metastasis, tumor stage, and its higher levels in the more aggressive PAM50 molecular subtypes, this was not found in the CPTAC or TCGA datasets. Nonetheless, since it was not possible to control for co-founding effects in any of the approaches and since BiP mRNA levels in tumors may differ from protein levels, these results deserve further investigation in prospective studies.

Previously, we and others showed that BiP expression was higher in Tamoxifen- and Fulvestrant-resistant breast cancer cell lines [9,59,60,61]. Given that BiP positivity was associated with metastasis, we used a small cohort of treatment-naïve luminal-like breast cancer to further investigate how BiP positivity relates to endocrine response. To the best of our knowledge, this is the first study analyzing the effect of hormone therapy on BiP positivity in human breast cancer tissue. BiP expression after antiestrogen treatment was significantly increased (mostly in the nucleus) and associated with patient death and with a higher tumor stage, thus supporting its association with metastasis and more aggressive tumors. However, BiP positivity was not correlated with Ki67 index and did not significantly change after treatment, although its subcellular localization was significantly increased in the nucleus. BiP can be directed to the nucleus [62] where it may play a role in reducing DNA damage-induced apoptosis [63,64], while in amyotrophic lateral sclerosis, it binds to TDP-43 to prevent its misfolding and subsequent toxicity [65]. Moreover, BiP was shown to molecularly interact with ERα and to be required for gene transcription in the uterus, serving as a hub between ERα-independent and ERα-dependent estrogenic responses [12]. Therefore, we can speculate that a similar regulation may occur in breast cancer, as part of the acquired endocrine resistance process, which may explain its nuclear localization in the metastasis. Future studies in larger cohorts are needed to further validate these associations and better understand BiP regulation in the context of acquired endocrine resistance in breast cancer.

BiP has been described in the cell surface of breast cancer cells; however, its prognostic value remains controversial: cell surface expression was associated with good prognosis and response to chemotherapy in PR+ breast cancers [12] and triple-negative breast cancer [13,66]. On the other hand, cell membrane relocalization of BiP was related to increased proliferation and migration [5], as well as with the increased metastatic potential and stemness [67] of breast cancer cells while no prognostic value of BiP cell membrane expression was found in a cohort of stage II–III breast cancer [6]. Although BiP cell surface expression is of specific relevance as there are currently anti-BiP antibodies in pre-clinical trials [68] in our cohort, we only found one case with few cells showing this subcellular localization. This may be explained by the different techniques used to detect cell surface BiP, namely the epitope recognized by the antibody used in this study and/or the system used to amplify the signal. In addition, it was recently demonstrated that cell surface BiP was present in very low amounts and in only a subpopulation (10%) of MCF-7 cells [69]. Since we only analyzed a small luminal-like breast cancer cohort, the lack of BiP detection on cell membranes may be also explained by this characteristic.

## 5. Conclusions

The three independent research approaches used in this study showed that higher BiP protein levels are associated with worse outcomes and their detection holds prognostic potential for breast cancer. Meta-analysis of publicly available RNA-seq and proteomics datasets of human breast cancers disclosed, for the first time, an association of BiP differential expression with immune signatures and a differential expression of BiP mRNA according to breast cancer molecular subtypes. The systematic review and meta-analysis of the immunohistochemical detection of BIP identified for the first time that ER and HER2 are factors that contribute to BiP positivity. This meta-analysis also confirmed that BiP positivity is significantly associated with recurrence-free survival. The immunohistochemical detection of BiP in two small cohorts of luminal breast cancers disclosed a novel finding, the effect of endocrine treatment on BiP subcellular localization and confirmed that increased BiP expression is associated with metastasis and HER2 expression. Nevertheless, factors contributing to heterogeneity in the immunohistochemical determination of BiP positivity, such as the type of antibody, the cut-off for positivity, and the subcellular localization as well as confounding effects, need to be further explored. In summary, BiP/GPR-78 has been implicated in response to stress caused by nutrient deprivation, hypoxia, or resistance to chemotherapy in breast cancer. An active UPR thus provides a survival advantage to cancer cells over normal cells and the analysis presented in this manuscript showed the association between BiP expression and HER2 and basal molecular subtypes (usually more aggressive than luminal-like tumors), and its association with metastasis and short relapse-free survival highlights the prognostic potential of BiP for breast cancer as an indicator of poorer outcomes.

## Figures and Tables

**Table 1:** Association of BiP-High (BiP-H) and BiP-Low (BiP-L) groups in CPTAC dataset with clinical factors.

Clinical Attribute	Attribute Type	Statistical Test	*p*-Value	*q*-Value	Higher in
TOP2A Proteogenomic Status	Patient	Chi-squared Test	5.67 × 10^−14^	1.82 × 10^−12^	BiP-H
ERBB2 Proteogenomic Status	Patient	Chi-squared Test	8.17 × 10^−13^	1.31 × 10^−11^	BiP-L
TNBREAST CANCER Updated Clinical Status	Patient	Chi-squared Test	1.38 × 10^−10^	1.47 × 10^−9^	BiP-H
xCell Immune Score	Patient	Wilcoxon Test	8.45 × 10^−5^	5.41 × 10^−4^	BiP-H
ESTIMATE Immune Score	Patient	Wilcoxon Test	3.10 × 10^−4^	1.66 × 10^−3^	BiP-H
PAM50	Sample	Chi-squared Test	9.61 × 10^−4^	4.39 × 10^−3^	
CIBERSORT Absolute Score	Patient	Wilcoxon Test	3.10 × 10^−3^	0.0123	BiP-H
ER Updated Clinical Status	Patient	Chi-squared Test	3.46 × 10^−3^	0.0123	BiP-L
ESTIMATE TumorPurity	Patient	Wilcoxon Test	7.61 × 10^−3^	0.0244	BiP-L
PR Clinical Status	Patient	Chi-squared Test	0.012	0.035	BiP-L

**Table 2:** Overrepresented pathways in TCGA PanCancer Atlas dataset stratified by BiP-High (BiP-H) and BiP-Low (BiP-L) quartiles.

#term ID	Term Description	Genes Mapped	Enrichment Score	Direction	FDR	Method
KEGG PATHWAYS						
**hsa04141**	**Protein processing in the endoplasmic reticulum**	**43**	**399.369**	**BiP-H**	**3.47 × 10^−12^**	**ks**
**hsa05169**	**Epstein–Barr virus infection**	**40**	**363.869**	**BiP-H**	**1.94 × 10^−7^**	**ks**
**hsa04612**	**Antigen processing and presentation**	**21**	**493.643**	**BiP-H**	**1.97 × 10^−7^**	**ks**
**hsa04110**	**Cell cycle**	**29**	**409.514**	**BiP-H**	**8.80 × 10^−7^**	**ks**
**hsa04650**	**Natural killer cell-mediated cytotoxicity**	**26**	**390.086**	**BiP-H**	**1.91 × 10^−6^**	**ks**
**hsa05164**	**Influenza A**	**30**	**351.733**	**BiP-H**	**4.67 × 10^−6^**	**ks**
**hsa05332**	**Graft-versus-host disease**	**15**	**461.606**	**BiP-H**	**1.40 × 10^−5^**	**afc**
**hsa04940**	**Type I diabetes mellitus**	**13**	**479.743**	**BiP-H**	**2.75 × 10^−5^**	**afc**
**hsa04145**	**Phagosome**	**38**	**26.468**	**BiP-H**	**6.58 × 10^−5^**	**ks**
**hsa05152**	**Tuberculosis**	**28**	**285.612**	**BiP-H**	**6.58 × 10^−5^**	**ks**
hsa03050	Proteasome	14	420.107	BiP-H	0.00023	afc
hsa04142	Lysosome	22	26.693	BiP-H	0.0031	ks
hsa05020	Prion disease	42	125.948	BiP-H	0.0087	ks
REACTOME PATHWAYS						
**HSA-168249**	**Innate immune system**	**178**	**269.185**	**BiP-H**	**2.16 × 10^−24^**	**ks**
**HSA-6798695**	**Neutrophil degranulation**	**90**	**348.227**	**BiP-H**	**1.06 × 10^−19^**	**ks**
**HSA-1280218**	**Adaptive immune system**	**132**	**248.407**	**BiP-H**	**2.22 × 10^−14^**	**ks**
HSA-1280215	Cytokine Signaling in the immune system	149	234.508	BiP-H	5.12 × 10^−12^	ks
HSA-913531	Interferon signaling	47	415.029	BiP-H	6.46 × 10^−12^	ks
HSA-1236975	Antigen processing cross-presentation	31	421.988	BiP-H	1.09 × 10^−9^	ks
HSA-909733	Interferon alpha/beta signaling	27	501.242	BiP-H	2.01 × 10^−9^	ks
HSA-72766	Translation	23	42.642	BiP-H	4.59 × 10^−9^	ks
HSA-1236974	ER–phagosome pathway	28	420.465	BiP-H	1.62 × 10^−8^	ks
HSA-983169	Class I MHC-mediated antigen processing and presentation	54	311.864	BiP-H	1.68 × 10^−8^	ks
HSA-5688426	Deubiquitination	45	299.335	BiP-H	3.27 × 10^−8^	ks
HSA-381119	Unfolded protein response (UPR)	20	437.966	BiP-H	8.16 × 10^−7^	afc
HSA-2132295	MHC class II antigen presentation	31	321.922	BiP-H	1.24 × 10^−5^	ks
HSA-381070	IRE1alpha activates chaperones	14	459.057	BiP-H	1.24 × 10^−5^	afc
HSA-449147	Signaling by Interleukins	97	186.579	BiP-H	1.79 × 10^−5^	ks
HSA-381038	XBP1(S) activates chaperone genes	13	428.195	BiP-H	0.00017	afc
HSA-977225	Amyloid fiber formation	13	330.029	BiP-H	0.0055	afc
HSA-983168	Antigen processing: ubiquitination and proteasome degradation	32	217.782	BiP-H	0.0060	ks

Bold denotes top10 pathway. ks: Kolmogorov–Smirnov test; afc: aggregate fold change.

**Table 3:** Overrepresented pathways in CPTAC protein dataset stratified by BiP-High (BiP-H) and BiP-Low (BiP-L) quartiles.

#term ID	Term Description	Genes Mapped	Enrichment Score	Direction	FDR	Method
KEGG PATHWAYS						
**hsa04141**	**Protein processing in the endoplasmic reticulum**	**40**	**183.735**	**BiP-H**	**9.15 × 10^−6^**	**ks**
**hsa04657**	**IL-17 signaling pathway**	**15**	**325.286**	**BiP-H**	**0.00014**	**afc**
**hsa01100**	**Metabolic pathways**	**151**	**0.503067**	**Both**	**0.0049**	**ks**
REACTOME PATHWAYS						
**HSA-6798695**	**Neutrophil degranulation**	**94**	**23.212**	**BiP-H**	**1.65 × 10^−9^**	**ks**
**HSA-168249**	**Innate immune system**	**170**	**156.502**	**BiP-H**	**3.49 × 10^−8^**	**ks**
**HSA-1474244**	**Extracellular matrix organization**	**37**	**249.065**	**BiP-H**	**1.52 × 10^−7^**	**ks**
**HSA-6799990**	**Metal sequestration by antimicrobial proteins**	**5**	**737.368**	**BiP-H**	**2.17 × 10^−6^**	**afc**
**HSA-6803157**	**Antimicrobial peptides**	**12**	**5.985**	**BiP-H**	**2.17 × 10^−6^**	**afc**
**HSA-72766**	**Translation**	**58**	**113.467**	**BiP-H**	**1.46 × 10^−5^**	**ks**
**HSA-1799339**	**SRP-dependent cotranslational protein targeting to membrane**	**46**	**119.151**	**BiP-H**	**0.00011**	**ks**
**HSA-1474228**	**Degradation of the extracellular matrix**	**16**	**280.973**	**BiP-H**	**0.00041**	**afc**
**HSA-71291**	**Metabolism of amino acids and derivatives**	**75**	**102.951**	**BiP-H**	**0.00041**	**ks**
**HSA-1442490**	**Collagen degradation**	**10**	**321.766**	**BiP-H**	**0.0020**	**afc**
HSA-877300	Interferon gamma signaling	18	222.754	BiP-H	0.0063	afc
HSA-381119	Unfolded protein response (UPR)	18	21.652	BiP-H	0.0077	afc
HSA-1280215	Cytokine signaling in the immune system	80	115.149	BiP-H	0.0089	ks
HSA-198933	Immunoregulatory interactions between a lymphoid and a non-lymphoid cell	13	248.601	BiP-H	0.0089	afc
HSA-1236975	Antigen processing cross-presentation	30	159.939	BiP-H	0.0095	ks
HSA-1280218	Adaptive immune system	103	0.869687	BiP-H	0.0098	ks
**HSA-5617833**	**Cilium assembly**	**32**	**251172**	**BiP-L**	**1.85 × 10^−5^**	**ks**
**HSA-1852241**	**Organelle biogenesis and maintenance**	**38**	**211028**	**BiP-L**	**0.00019**	**ks**
**HSA-73894**	**DNA repair**	**31**	**198671**	**BiP-L**	**0.00099**	**ks**
**HSA-9018519**	**Estrogen-dependent gene expression**	**15**	**328834**	**BiP-L**	**0.0020**	**afc**
**HSA-74160**	**Gene expression (Transcription)**	**111**	**0.735739**	**BiP-L**	**0.0050**	**ks**
HSA-5620924	Intraflagellar transport	16	296691	BiP-L	0.0060	afc

Bold denotes the top ten pathways. ks: Kolmogorov–Smirnov test; afc: aggregate fold change.

**Table 6:** Meta regression summary table.

Stratification	No. of Studies	*p*-Value	I2 (%)
Menopause status	6	0.1092	70.00
Tumor stage	7	0.7248	75.84
Tumor grade	9	0.0498 *	74.77
Lymph node metastasis	10	0.9594	84.40
ER expression	10	0.0371 *	68.72
HER2 expression	11	<0.0001 *	47.08
Age	5	0.0018 *	0.00

* Significative association.

**Table 7:** Correlation between BiP expression clinicopathological factors in cohort 1.

Clinical Factor	Cases, No.	*p*-Value
Histology	14	0.449
Age	14	1.000
Grade	14	0.014 *
HER2+	14	0.046 *
Stage	14	0.946
Lymph node metastasis	14	0.308
Vital status	14	0.353
Response to HT	14	0.120
Ki-67 index	14	0.373 ^#^

* Statistically significant Fisher exact test. ^#^ Pearson’s correlation test.

**Table 8:** Correlation between BiP subcellular localization and clinical factors in IPOP’s cohort 1.

Clinical Factor	Cases, No.	*p*-Value
HER2+	14	1.000
Grade	14	0.209
Stage	14	0.038 *
Lymph node metastasis	14	0.209
Vital status	14	0.043 *
Response to HT	14	0.326

* Statistically significant Fisher exact test.

**Table 9:** Correlation between BiP expression and the clinicopathological factors in cohort 2.

Clinical Factor	Cases, No.	*p*-Value
Age	12	1.000
Histology	12	0.510
Grade	12	0.567
HER2+	12	1.000
Stage	11	1.000
Lymph node metastasis	8	1.000
Time to metastasis	12	0.834

**Table 10:** Correlation between BiP subcellular localization and clinical factors in cohort 2.

Clinical Factor	Cases, No.	*p*-Value
HER2+	12	1.000
Grade	12	1.000
Stage	11	0.900
Lymph node metastasis	8	0.604
Time to metastasis	12	0.682

## Data Availability

The data presented in this study are available in the article and supplementary material. The TCGAPanCancer and CPTAC dataset are publicly available through: https://www.cbioportal.org/ (accessed on 22 July 2022).

## Supplementary Materials

The following supporting information can be downloaded at: https://www.mdpi.com/article/10.3390/curroncol29120710/s1, Table S1: List of causes for excluding studies from the metanalysis, Table S2: Clinicopathological data of the patient cohort 1 from IPOP, Table S3: Clinicopathological data of the patient cohort 2 from IPOP.
