# Supplementary material for: The Clinicopathological Significance of BiP/GRP-78 in Breast Cancer: A Meta-Analysis of Public Datasets and Immunohistochemical Detection
Source: Curr Oncol. 2022 Nov 23;29(12):9066–87. doi: 10.3390/curroncol29120710 (PMC9777260; doi:10.3390/curroncol29120710)
Supplement: Supplementary file 1 [file curroncol-29-00710-s001.zip › Supplementary table S2-cohort 1 characteristics_VF.pdf]

Supplementary table S2 - Clinicopathological data of the patient cohort 1 from IPOP

Patient	Age at diagnosis	Histological type	Grade	Stage	Molecular subtype	Adjuvant Treatment	Metastasis topography	Disease course
1	49	Lobular	2	III	Luminal B-like, HER2+	CT + RT + Letrozol + Anastrazol	N/A	Alive without disease
2	62	Ductal	2	IV	Luminal B-like, HER2-	CT + Letrozol	N/A	Alive without disease
3	81	Ductal	1	II	Luminal-like, HER2-	Exemestan	N/A	Alive without disease
4	48	Lobular	2	N/A	Luminal-like, HER2-	CT + HT (not specified)	Multiple metastasis	Dead of disease (metastasis)
5	40	Neuroendocrine differentiation	3	IV	Luminal-like, HER2-	CT + RT + Exemestan + Fulvestrant + Letrozol	Skin, Liver	Dead of disease (metastasis)
6	76	Ductal	1	III	Luminal-like, HER2-	RT + Letrozol + Tamoxifen	N/A	Alive with disease
7	84	Ductal	2	III	Luminal-like, HER2-	RT + Letrozol	N/A	Alive without disease
8	77	Ductal	2	II	Luminal-like, HER2-	RT+ Letrozol	N/A	Alive without disease
9	69	Mixed	1	III	Luminal-like, HER2-	RT+ Letrozol	N/A	Alive without disease
10	68	Ductal	3	III	Luminal-like, HER2+	CT + Anastrazole + RT + Tamoxifen + Docetaxel + Fulvestrant + Palbociclib	Bone	Dead of disease (metastasis)
11	75	Ductal	1	IV	Luminal-like, HER2-	Letrozol + RT + Palbociclib	N/A	Alive without disease
12	83	Ductal	2	II	Luminal-like, HER2-	RT + Anastrazole	N/A	Alive without disease
13	74	Ductal	2	II	HER2-	RT+ Letrozol	N/A	Alive without disease
14	87	Ductal	3	III	HER2+	Anastrazole	N/A	Alive without disease

Abbreviations: CT – chemotherapy; N/A – not available; RT – radiotherapy; HT – Hormonal therapy
