# Supplementary material for: The Clinicopathological Significance of BiP/GRP-78 in Breast Cancer: A Meta-Analysis of Public Datasets and Immunohistochemical Detection
Source: Curr Oncol. 2022 Nov 23;29(12):9066–87. doi: 10.3390/curroncol29120710 (PMC9777260; doi:10.3390/curroncol29120710)
Supplement: Supplementary file 1 [file curroncol-29-00710-s001.zip › Supplementary table S3 - cohort 2 characteristics_VF.pdf]

Supplementary table S3 - Clinicopathological data of the patient cohort 2 from IPOP

Patient	Age at diagnosis	Histological type	Grade	Stage	Molecular subtype	Adjuvant Treatment	Years until metastasis	Metastasis topography	Disease course	Vital Status
BCM001	30	Ductal	2	III	Luminal B-like, HER2+	CT + RT + Tamoxifen + Trastuzumab	11	Brain	Disease progression, under endocrine treatment (bone, adrenal, lung and ovarian metastases)	Dead of disease
BCM004	36	Mixed	2	III	Luminal B-like, HER2+	CT + RT + Exemestane + Trastuzumab	10	Brain	Disease progression, under endocrine treatment (skin, bone metastases)	Dead of disease
BCM080	56	Ductal	3	II	Luminal-like, HER2-	CT + RT + Anastrazole	8	Skin	Disease progression (bone metastases)	Dead of disease
BRT3DP	59	Ductal	3	N/A	Luminal-like, HER2+	CT + RT + Tamoxifen	6	Pleural	Disease progression, under endocrine treatment (bone, skin, lymph-node metastases)	Dead of disease
BRT5DP	67	Lobular	2	II	Luminal-like, HER2-	CT + RT + Anastrazole	8	Pleural	Disease progression (bone metastases)	Dead of disease
BRT6DP	68	Lobular	2	II	Luminal-like, HER2-	CT + RT + Tamoxifen→Exemestane	8	Pleural	Disease progression, under endocrine treatment (bone, liver metastases)	Dead of disease
BRT15DP	44	Ductal	3	III	Luminal-like, HER2-	CT + RT + Tamoxifen	5	Pleural	Disease progression, under endocrine treatment (bone, brain, liver,	Dead of disease

									lymph-node metastases)	
BRT29DP	45	Ductal	2	III	Luminal-like, HER2-	CT + RT + Tamoxifen	5	Pleural	Disease progression (bone, lymph-node, liver, skin metastases)	Dead of disease
BRT41DP	66	Ductal	3	III	Luminal-like, HER2+	CT + Anastrazole + Trastuzumab	2	Pleural	Disease progression (lung metastases)	Dead of disease
BRT56DP	54	Ductal	3	I	Luminal-like, HER2-	CT + Anastrazole	5	Pleural	Disease progression, under endocrine treatment (lung, pericardium, bone, lymph-node metastases)	Dead of disease
BRT57DP	51	Lobular	2	I	Luminal-like, HER2-	CT + Tamoxifen	2	Pleural	Disease progression, under endocrine treatment (bone, liver metastases)	Dead of disease
BRT67DP	69	Ductal	3	III	Luminal-like, HER2-	CT + RT + Anastrazole	4	Pleural	N/A	Died of stage IV gastric cancer

Abbreviations: CT – chemotherapy; N/A – not available; RT - radiotherapy
